# Application of Citicoline in Neurological Disorders: A Systematic Review

**DOI:** 10.3390/nu12103113

**Published:** 2020-10-12

**Authors:** Patryk Jasielski, Faustyna Piędel, Mikołaj Piwek, Agata Rocka, Véronique Petit, Konrad Rejdak

**Affiliations:** Department of Neurology, Medical University of Lublin, 20-059 Lublin, Poland; faustyna.piedel@gmail.com (F.P.); miki2398@gmail.com (M.P.); agatarocka2@gmail.com (A.R.); vpetit@onet.pl (V.P.); konrad.rejdak@umlub.pl (K.R.)

**Keywords:** citicoline, neurology, supplementation, treatment

## Abstract

Citicoline is a chemical compound involved in the synthesis of cell membranes. It also has other, not yet explained functions. Research on the use of citicoline is conducted in neurology, ophthalmology, and psychiatry. Citicoline is widely available as a dietary supplement. It is often used to enhance cognitive functions. In our article, accessible databases were searched for articles regarding citicoline use in neurological diseases. This article has a systemic review form. After rejecting non-eligible reports, 47 remaining articles were reviewed. The review found that citicoline has been proven to be a useful compound in preventing dementia progression. It also enhances cognitive functions among healthy individuals and improves prognosis after stroke. In an animal model of nerve damage and neuropathy, citicoline stimulated regeneration and lessened pain. Among patients who underwent brain trauma, citicoline has an unclear clinical effect. Citicoline has a wide range of effects and could be an essential substance in the treatment of many neurological diseases. Its positive impact on learning and cognitive functions among the healthy population is also worth noting.

## 1. Introduction

Citicoline is an abbreviation of cytidine-5′-diphosphocholine (CDP-choline). It is an endogenous chemical compound. Citicoline is globally available as a dietary supplement and in many countries as a drug. It can be bought as a tablet or an injection. In the human body, citicoline is degraded to cytidine and choline during hydrolysis and dephosphorylation. Subsequently, cytidine and choline are substrates for phosphatidylcholine and CDP-choline synthesis in neurons. However, the detailed mechanism of citicoline functioning is not well understood [1,2]. Citicoline has minimal toxicity and is rapidly metabolized. Products of metabolization are eliminated as carbon dioxide. Citicoline safety has been repeatedly proven in research based on animals [3].

Citicoline has comprehensive neuroprotective properties. One such mechanisms is an increase in sirtuin-1 level (silent information regulator 1, SIRT1). SIRT1 belongs to the histone deacetylase family. SIRT1 regulates metabolic homeostasis and neuronal aging [4]. It may also have neuroprotective effects and have a beneficial effect on neurodegenerative diseases such as Parkinson’s and Alzheimer’s diseases [5,6,7]. Citicoline raises the level and increases SIRT1 activity in the rat brain, cultured neurons, and in peripheral blood mononuclear cells [8].

Another mechanism is related to the influence on the levels of neurotransmitters in synapses. Citicoline increases the level of dopamine and norepinephrine in the central nervous system, which contributes to neuroprotection in hypoxia [9]. Choline, one of the breakdown products of citicoline, serves as a substrate for the synthesis of acetylcholine. This neurotransmitter also has a neuroprotective effect [10,11]. Citicoline raises the level of serotonin, which is also supposed to promote neuroprotective effects [12]. Citicoline lowers glutamate levels. This neurotransmitter, mainly through the action of the N-methyl-d-aspartate (NMDA) receptor, is responsible for damage to the brain during ischemia [13]. Citicoline is an intermediary for the synthesis of phosphatidylcholine, which is composed of the neuronal cell membrane. Thus, it has neuroprotective properties because a greater availability of phosphatidylcholine may stimulate the repair and regeneration of damaged cell membranes of neurons [12,14,15]. Moreover, when choline is depleted, phospholipids are hydrolyzed to restore choline levels. Acetylcholine synthesis is favored when the available amount of choline is limited. Therefore, citicoline is a source of choline, avoiding phosphatidylcholine hydrolysis [16].

Another likely mechanism of action is to block inflammation (caused by, e.g., ischemia) by inhibiting phospholipase A2. This enzyme is involved in the breakdown of membrane phospholipids into arachidonic acid. The oxidative metabolism of arachidonic acid contributes to the generation of neuroinflammation and reactive oxygen species (ROS). By blocking phospholipase A2, citicoline may contribute to the reduction of inflammation, ROS formation, and neuronal damage [17]. Citicoline may also show anti-apoptotic effects [18]. Citicoline is also beneficial in glaucoma and amblyopia [1,2]. Research on animals and humans demonstrated that citicoline improves brain functions and stunts cognitive deficits [19].

Concluding, in research based on animals and humans, it was proved that citicoline is beneficial in the regeneration of neurons, can increase levels of neurotransmitters, and has a positive impact on cognitive functions. Moreover, it can be an additional drug in the therapy of depression and mood regulation.

## 2. Materials and Methods

A search for articles about the usage of citicoline in the treatment of neurological disorders was performed. The following databases were analyzed: PubMed, Scopus, Web of Science, Cochrane Library, and Clinicaltrials.gov. The search was undertaken in April 2020. To find articles, the following keywords were used: “citicoline”, “neurology disorders”, “CDP-choline”, and “cytidine-5′-diphosphocholine”. A three-step analysis of found articles—title, abstract, and entire text—was undertaken. Two independent scholars conducted the analysis. Inclusion and exclusion criteria are summarized in the table (Table 1). Research based on animals is included in only one subsection about neuropathic pain and neuronal regeneration.

**Figure 1:**
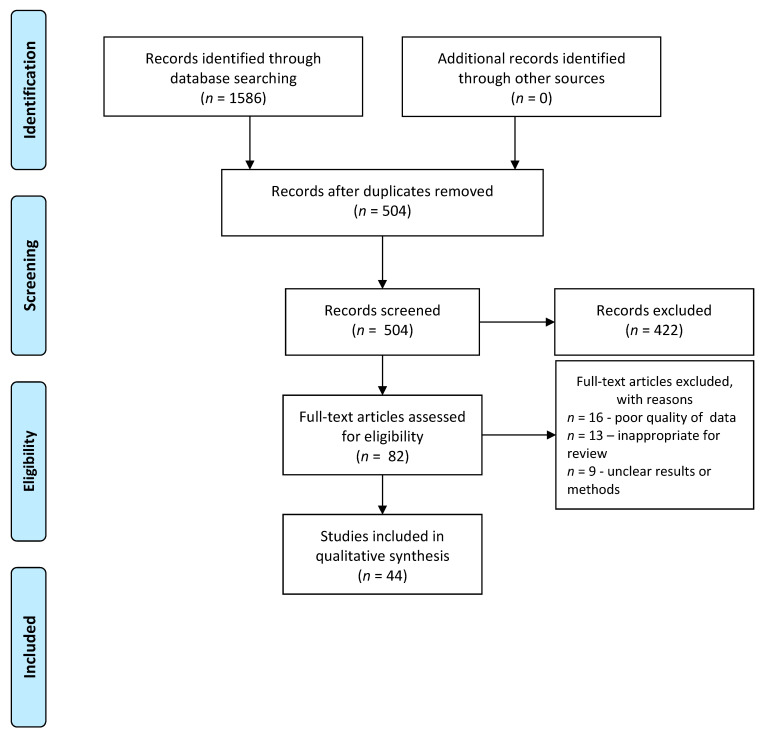
Preferred Reporting Items for Systematic Reviews and Meta-Analyses (PRISMA) flowchart.

The summary of articles analyzed is presented on the Preferred Reporting Items for Systematic Reviews and Meta-Analyses (PRISMA) flowchart (Figure 1).

## 3. Results

A total of 504 records were screened after the implementation of inclusion criteria. After assessment for eligibility, 47 studies were included in the qualitative synthesis.

### 3.1. Application of Citicoline in Brain Stroke

Stroke is one of the most common neurological conditions. Depending on its location and size, it can lead to severe neurological disorders or be practically asymptomatic. The application of appropriate rehabilitation, support, and neuroprotective treatment may reduce its harmful effects and facilitate recovery, or at least amend the functioning of the patient [20]. The following scales are used to assess the effectiveness of the applied therapy: Barthel index (BI), National Institute of Health Stroke Scale (NIHSS), and modified Rankin Scale (mRS). The NIHSS and mRS scales are used to assess body dysfunction in patients who have suffered a stroke. The higher the score on both scales, the greater the assessed organism dysfunction and worse functioning [21,22]. The BI scale is used to assess everyday functioning, and ability to cope with basic tasks (including eating, dressing, taking care of hygiene). The higher the result on the BI scale, the better the respondent’s functioning [23].

Mehta et al. administrated neuroprotective drugs—citicoline, edaravone, minocycline, and cerebrolysin—in patients with ischemic stroke of the middle cerebral artery. In the citicoline group, the NIHSS score at baseline was 14.00 ± 4.34, whereas after 11 days, it was 8.90, and after 90 days, it was 3.53. In both measurements, these results were significant compared to the control group (*p* < 0.001). In the BI scale, significant results were also obtained (*p* < 0.001): at the beginning of the therapy, the value was 36.0; on day 11, it was 64.0; and at 90 days, it was 86.0 [24]. Other researchers obtained similar positive results of citicoline. During the 3-month follow-up, stroke patients were divided into the citicoline group and the control group. Patients treated with citicoline achieved significantly higher BI scores both in the 1st and 3rd month (*p* < 0.001 in 1st month, *p* = 0.002 in 3rd month). The obtained results were more favorable in the study group, both in patients after ischemic and hemorrhagic stroke [25]. The advantage of the following study is its longer period of stroke patient observation. Patients received citicoline for one year, 6 weeks after a diagnosed stroke. In the clinical evaluation, both after 6 and 12 months, no significant differences were noticed in the results obtained on the mRS scale by the control group and the group receiving citicoline (*p* = 0.186). However, patients receiving citicoline achieved better results in terms of cognitive functions: attention–executive functions (odds ratio (OR) 1.721, 95% confidence interval (CI) 1.065–2.781, *p* = 0.027 at 6 months; OR 2.379, 95% CI 1.269–4.462, *p* = 0.007 at 12 months) and temporal orientation (OR 1.780, 95% CI 1.020–3.104, *p* = 0.042 at 6 months; OR 2.155, 95% CI 1.017–4.566, *p* = 0.045 at 12 months), even if taking into account risk factors and the severity of the stroke [26]. In addition to clinical improvement, another study examined the effect of citicoline on the risk of death 90 days after stroke. The frequency of incidences of non-neurological complications during hospitalization was also analyzed. In the mRS assessment, patients receiving citicoline after 30 days had significantly lower mean scores and lower median scores (*p* = 0.03 for both). After 90 days, the difference between the groups was no longer statistically significant, while the trend of better results continued in the citicoline group. Adjusting for age, gender, NIHSS at hospital admittance, hospital arrival in <24 h, and relevant risk factors, citicoline treatment was independently associated with a lower 30- and 90-day mortality risk (OR 0.30, 95% CI 0.10–0.88, *p* = 0.03; OR 0.33, 95% CI 0.12–0.87, *p* = 0.03, respectively), and with a lower rate of non-neurological complications acquired during hospitalization (OR 0.20, 95% CI 0.08–0.22, *p* = 0.001) [27]. In the study by Alvarez-Sabín et al. [28], the scholars examined the effect of citicoline on the quality of life of people after ischemic stroke. To assess the quality of life, the EuroQoL-5D scale was used. Patients who received citicoline had a significantly better quality of life compared to the control group (*p* = 0.041). Citicoline use, regardless of the age of the respondents, was an independent factor improving the quality of life and was beneficial for patients after stroke. Moreover, the absence of citicoline treatment (OR 2.321, 95% CI 1.057–5.100, *p* = 0.036) was an independent predictor of poor or very poor quality of life. Additionally, the patients’ cognitive functions were examined at 1 month, 6 months, 1 year, and 2 years. After 2 years, patients receiving citicoline had fewer cognitive impairments, but this did not reach statistical significance. However, this group achieved a significant improvement in cognitive functions (*p* = 0.005). The control group did not obtain a significant amendment.

Cho et al. [29] conducted a study on a group of 4191 people. A total of 3736 patients received citicoline up to 24 h after stroke (early group) and 455 received treatment later than 24 h (late group). For clinical evaluation, a short form of the National Institute of Health Stroke Scale (s-NIHSS), a short form of the Barthel Index (s-BI), and mRS were used. S-NIHSS and s-BI at week 6 of citicoline administration were significantly better than at baseline. S-NIHSS improved from 9.8 ± 2.9 at baseline to 6.9 ± 2.4 at 6 weeks (*p* < 0.001), and s-BI improved from 6.0 ± 1.9 to 4.2 ± 1.7 (*p* < 0.001). In 125 patients who received citicoline for more than 12 weeks, further improvements were noted in s-NIHSS at the end of therapy compared to at week 6 (6.4 ± 1.6, *p* < 0.001). The early group showed significantly more improvement in s-NIHSS and s-BI at week 6 (mean s-NIHSS changes between baseline and 6 weeks: early group, 3.0 ± 2.2; late group, 2.1 ± 2.4; *p* < 0.001). After adjustment for age, sex, and risk factors, these results were significant for the s-NIHSS at week 6 (*p* < 0.001). Efficiency of the therapy was dose dependent. Improvements were more significant in the higher dose group (≥2000 mg/day) (*p* < 0.001).

Another study analyzed the effect of different citicoline doses on changes in the BI score using logistic regression analysis with the baseline NIHSS score used as a covariate. There was a significant treatment effect seen with the logistic regression analysis comparing citicoline treatments to placebo at 12 weeks (*p* ≤ 0.05). The OR for improvement in BI on citicoline 500 mg treatment was 2.0, and on 2000 mg, it was 2.1. The total mean BI scores for the four groups at 12 weeks were 56 for placebo, 71 for 500 mg, 55 for 1000 mg, and 65 for 2000 mg. The mean differences in the total BI scores were statistically significant (*p* ≤ 0.05) for the 500 mg group compared with the placebo group. Full recovery (BI ≥ 95) after 12 weeks was completed in 33% of patients from the placebo group, 53% from 500 mg, 29% from 1000 mg, and 45% from the 2000 mg group. There was a significant difference between placebo and 500 mg groups (*p* = 0.01). The mean mRS scores for the four groups were 3.1 for placebo, 2.5 for the 500 mg citicoline group, 3.1 for the 1000 mg citicoline group, and 2.6 for the 2000 mg citicoline group [30].

The next study assessed the effect of citicoline on the percentage of independent patients at 3 months using the mRS (independent when mRS score ≤ 2) scale in people after hemorrhagic stroke. Secondary efficacy endpoints included the evolution of neurological deficits assessed with the NIHSS. After 3 months, 5 patients from the citicoline group attained mRS ≤ 2; in the control group, only 1 patient attained this standard (OR 5.38, 95% CI 0.55–52.4). The evolution of NIHSS scores from baseline to 12 weeks were similar and reached statistical significance in both groups (*p* < 0.01) [31].

In another analyzed study, the Scandinavian Stroke Scale (SSS) was used to assess neurological deficits in patients receiving and not receiving citicoline. This scale includes both the prognostic score (which assesses, among other aspects, awareness) and also the long-term score (which determines, among other factors, limbs muscle strength, speech, and gait). A higher score on this scale indicates lower neurological deficits. In the conducted study, differences were noticed on the 3rd day after starting citicoline therapy. In terms of orientation and awareness, 90.1% of the respondents receiving citicoline were fully aware and oriented; while in the control group, only 74.5% were fully aware and oriented (OR 3.12, 95% CI 1.12–8.77, *p* = 0.026). Patients treated with citicoline also had faster improvement in speech. On day 7, no significant differences were noticed in terms of neurological deficits between the groups in the SSS scale (results 41.1 in the study group and 39.1 in the control group). However, on day 21 of follow-up, patients in the citicoline group achieved significantly better SSS scores (49.2 and 44.7 points, respectively, *p* = 0.017). The improvement was noticeable, especially in terms of mobility of the lower limbs (*p* = 0.036) and in gait (*p* = 0.002). The results obtained in the BI and mRS scale on the day of discharge from the hospital (day 21–24) were also better in the research group. The effects on the BI scale were 89.9, while patients in the control group were 82.3, *p* = 0.039. Moreover, in the group treated with citicoline, a significantly higher percentage of patients obtained the result of a complete return to the ability to perform daily activities (BI > 90) (OR 4.01, 95% CI 1.73–9.37, *p* = 0.006). The reference group included more patients with unsatisfactory recovery, i.e., with total points scores of ≤60 (OR 3.48, 95% CI 1.37–8.95, *p* = 0.013). In addition, on the mRS scale, patients from the research group obtained better results: 54 did not have significant symptoms and were able to perform everyday duties, whereas such an outcome was obtained by 17 people from the control group (OR 4.01, 95% CI 1.73–9.37, *p* = 0.0006) [32].

Iranmanesh et al. [33] assessed the effect of citicoline on muscle strength in patients after hemorrhagic stroke. Muscular strength was assessed using the Lovett scale. The mean muscular strength before intervention in all patients was 2.5 (1–4). After treatment, in the citicoline group, the mean muscle force increased to 4 (1–5), and in the placebo group, it increased to 3.12 (1.5–5) (Mann–Whitney test, *p* = 0.019). Citicoline helped to restore muscle strength to a greater extent than placebo.

The Japanese Coma Scale (JCS) and Global Improvement Rating (GIR) were used in another study. GIR was assessed as six categories based on changes in the level of consciousness, individual neurologic signs, and the patient’s general condition. Improvements in the level of consciousness were similar between groups on days 1, 2, and 3, but they were significantly improved for the citicoline group on days 7 and 14. The rates of improvement were calculated at the final assessment: 51% for the citicoline group compared with 33% in control group (*p* < 0.05). On Days 2, 7, and 14, the GIR of the citicoline group was significantly better than that of the placebo group. Improvement was noted in 32% and 18% on Day 7, and 54% and 29% on Day 14, for the citicoline and placebo groups, respectively. Rates of improvement at the final assessment were 52% for patients receiving citicoline and 26% for those receiving placebo (*p* < 0.01) [34].

In a study by Sobrino et al. [35], the influence of citicoline on the number of circulating endothelial progenitor cells (EPCs) was assessed in correlation with the improvement of the health assessed by NIHSS and mRS scales. The level of EPCs is associated with cardiovascular risk. Higher concentrations of these cells indicate a better prognosis. Some of the patients receiving citicoline were also treated with recombinant plasminogen activator (14 patients, 12 without such treatment). An increase in the number of colony-forming unit–endothelial cell (CFU-EC) units 1 week after the start of treatment was considered an indicator of a high probability of good treatment outcomes. Such an increase was significantly more frequent (*p* < 0.0001) among those receiving citicoline alone as compared to in combination with thrombolytic therapy. In the model, after taking into account the size of the stroke and the time from the onset of stroke, the administration of citicoline (OR 17.6, 95% CI 2.3–137.5, *p* = 0.006) and citicoline with thrombolytic drugs (OR 108.5, 95% CI 2.9–1094.2, *p* = 0.001) were independently associated with an increase in CFU-EC ≥ 4. After 3 months, the results obtained in the NIHSS (*p* = 0.003) and mRS (*p* = 0.012) scales were significantly better in the group receiving citicoline, both alone and with a thrombolytic drug. In addition, the amount of CFU-EC in this group after 3 months was significantly higher than that in the control group (*p* < 0.0001).

Another study investigated the effect of citicoline administration on the levels of angiostatin, neurofilaments, and acid fibril protein in people after an ischemic stroke associated with atrial fibrillation. Acid fibril protein is a sensitive marker of brain tissue damage and the effectiveness of neuroprotection. In humans, the concentration of this protein correlates with the formation of the astrocytic scar after trauma or stroke. Neurofilaments are considered a marker of neuronal death and may be useful in determining the prognosis after stroke. The last substance to be tested was angiostatin, which is an anti-angiogenic substance. However, the role of this substance in ischemic stroke is still unclear. In the citicoline group, the levels of both neurofilaments and acid fibril protein were significantly lower (*p* < 0.05 and *p* < 0.01, respectively). In addition, the level of angiostatin decreased to a significant extent (by 40% to the baseline value) (*p* < 0.05). In the control group, there was no change in the level of the tested substances in relation to the base concentration. On this basis, the authors concluded that citicoline has a protective effect on astrocytes and neurons. Moreover, it has a beneficial impact on the regulation of angiogenesis in an ischemically damaged brain [36].

In the next study, patients with hemorrhagic stroke took part. The influence of citicoline on the level of acid fibril protein, and on the level of copeptin, was also assessed. Copeptin is a glycoprotein that is thought to be a prognostic factor in stroke. After 28 days of treatment, both the levels of fibril acid and copeptin in the citicoline group were significantly lower than in the control group (both *p* < 0.05). Patients were also assessed using the NIHSS and BI scales. After 28 days, the NIHSS result in the research group was 9.43, while in the control group, it was 14.56 (*p* < 0.05). Similarly, on the BI scale, people from the research group obtained better results (69.28 vs. 51.57) (*p* < 0.05). In both groups, all the parameters tested had a similar value at the beginning of the experiment [37].

Another potential effect of citicoline in ischemic stroke may be its effect on cerebral perfusion. At the beginning of the treatment, patients underwent transcranial and extracranial Doppler ultrasound examinations of cerebral circulation. The flow in the following arteries was assessed: common carotid, internal carotid, anterior, middle and posterior brain, ocular, vertebral, and basilar arteries. There were significant differences between the groups in the maximum systolic velocity and the mean flow velocity. The maximum systolic velocity differed significantly in the following arteries: right common carotid (*p* = 0.008), internal carotid (*p* = 0.031), right (*p* = 0.008) and left (*p* = 0.002) vertebral arteries; in all of these arteries, the velocities measured were lower in the citicoline group. The mean flow velocity was significantly different between the right internal carotid (*p* = 0.031) and left anterior cerebral artery groups (*p* = 0.033); the measured speeds were also lower in the citicoline group. The study groups, with the exception of the supply of citicoline, did not differ in clinical features. This study was the first of its kind to investigate the effects of citicoline on cerebral perfusion. The clinical significance of these differences is not fully understood. Further research is recommended [38].

In a meta-analysis by Secades et al. [39], ten studies were included, in which a total of 4420 patients participated. The primary efficacy measure was patient independence at the end of the scheduled follow-up. For this purpose, the mRS scale was used (mRS score of 0–2 indicates independence of patient). In the studies where mRS was not available, the most comprehensive measure of disability from trial was used. The administration of citicoline was associated with a significant higher rate of independence, regardless of the method of evaluation (OR 1.56, 95% CI 1.12–2.16 under random effects; OR 1.20, 95% CI 1.06–1.36 under fixed effects). The time gap between studies is 32 years. Hence, a significant level of heterogeneity was observed (*p* = 0.0002).

All of the analyzed articles noted thus far indicated that citicoline is an effective drug in the improvement of the clinical condition of patients after stroke. However, based on subsequent articles, it can be concluded that the decisive role of citicoline is uncertain.

In a multicenter study called ICTUS, in which over 2000 patients participated, more than 1000 received citicoline for 6 weeks after an ischemic stroke event. A total of 873 were included in the protocol (the rest died or did not meet the requirements). Ninety days after the start of treatment, patients enrolled in the protocol were re-assessed using the NIHSS, mRS, and BI scales. After this period, the general state of health was similar in both groups (OR 1.03, 95% CI 0.86–1.25; *p* = 0.364). After 90 days, mRS ≤ 1 was achieved by 169 (19.4%) subjects in the citicoline group and 163 (19.2%) subjects in the placebo group (OR 1.04, 95% CI 0.79–1.36). On the NIHSS scale, the result of ≤1 in the study group was obtained by 209 (24.1%) patients, and in the control group, the result of ≤1 was achieved by 190 patients (22.3%) (OR 1.17, 95% CI 0.91–1.51). In BI, the result of ≥95 was obtained in the group receiving citicoline by 250 (28.8%) of the patients, and in the control group, it was obtained by 246 patients (28.9%) (OR 1.01, 95% CI 0.79–1.28). The obtained mRS results were similar for both groups. In the group covered by the protocol, the mean raw improvement in the NIHSS scale in the research group was 2.18, and in the control group, it was 0.91 (*p* = 0.051). However, in the group of people over 70 years of age, citicoline was found to have more beneficial properties for functioning than in the younger group of patients (OR 1.17, 95% CI 0.92–1.50, *p* = 0.001). The same relationship was observed for patients with an NIHSS score < 14 (OR 1.08, 95% CI 0.86–1.35, *p* = 0.021) and for patients not treated with thrombolytic therapy (OR 1.11, 95% CI 0.85–1.46, *p* = 0.041). Authors included an interpretation of their study and an updated meta-analysis consisting of six clinical trials. When the best treatment was applied, citicoline did not display any clinical improvement. Nevertheless, the effect of the drug remains significant (OR 1.14, 95% CI 1.00–1.30), which is based on an updated fixed-effects meta-analysis. Moreover, in the meta-analysis, a significant heterogeneity of effects (*p* = 0.0029) between the previous studies and the ICTUS trial was observed. Beneficial properties of citicoline for patients over 70 years of age, who did not receive thrombolytic therapy and with an NIHSS score < 14, and a lack of these positive outcomes in other groups, require further analyses. Mortality and the frequency of side effects were similar in both groups [40]. Another study tested the effectiveness of citicoline and edaravone in patients after ischemic stroke. Three months after starting treatment, the patients’ condition was assessed using the NIHSS and mRS scales. Patients receiving citicoline had slightly better results than the control group on the mRS scale (1.95 vs. 2.08) and also on the NIHSS scale (6.41 vs. 7.08). However, these differences were not significant [41].

The meta-analyses mentioned before [39,40] were analyzed by Yu et al. [42]. The authors used a parametric analysis of meta-analyses, maximum likelihood estimate (MLE), which can be interpreted as a weighted average of the study-specific estimate of the effect size with a shrinkage and Bayes estimator for standardized mean difference. The authors presented also a method to account for publication bias. Using their methods, the researchers obtained slightly lower OR values than in previous meta-analyses. However, the obtained OR values still remained statistically significant. OR for the meta-analysis by Secades et al. was 1.10, 95% CI, 1.02–1.17, and for study ICTUS, it was OR 1.08, 95% CI, 1.01–1.16.

Clark et al. [43] used BI and NIHSS to assess the effectiveness of citicoline. Unfortunately, after 12 weeks, no significant differences between the control and citicoline group were observed (*p* > 0.05). In patients with mild baseline strokes (NIHSS < 8), no differences were seen between groups. However, for patients with moderate-to-severe strokes (NIHSS ≥ 8), citicoline treatment was beneficial. In this group, BI ≥ 95 was attained by 33% vs. 21% in the control (*p* = 0.05), mRS ≤ 1; 19% vs. 11% (*p* = 0.07), and NIHSS ≤ 1; 19% vs. 11% (*p* = 0.08). In this group, citicoline treatment appears to be an overall benefit (OR 1.9, *p* = 0.04). Moreover, the percentage of patients who had large improvements in their NIHSS scores (≥7) at 12 weeks compared with the baseline was evaluated. A higher percentage (42%) of citicoline-treated patients had improvement compared with patients receiving placebo (30%) (*p* = 0.01). Similar results were obtained in the next study [44]. After 90 days, no significant differences were observed between the citicoline and placebo groups (52% vs. 51% patients attained NIHSS ≥ 7). Furthermore, there were no between-group differences in mortality.

Another study assessed changes in the volume of ischemic lesions using magnetic resonance imaging (MRI) and changes in clinical condition. The primary statistical analysis of the difference in the distribution of changes in lesion volume between placebo and citicoline groups from baseline to week 12 by the Smirnov test was not significant (*p* = 0.18). In patients who were treated 12 h or less from stroke onset, a larger growth of the lesion (mean ± SE) between baseline and week 12 was observed in the placebo group than in the citicoline group (30.3 ± 11.4 craniocaudal (cc) vs. 10.5 ± 6.0 cc planes, respectively). However, there were no significant differences between treatment groups in any of the clinical outcome measures. Citicoline did not significantly improve either clinical outcome or brain lesion in MRI [45].

Based on the above analysis, it is not possible to unequivocally state whether citicoline is an effective drug improving the clinical condition of people after stroke. In the vast majority of studies, citicoline had a significant beneficial effect both on the clinical situation and on molecular changes. However, one sizeable multicenter study undermined the efficacy of citicoline and showed its uncertain effectiveness. Further research is required to determine the effectiveness and potential mechanisms of the action of citicoline. The most important features of the research cited above are summarized in the table (Table 2).

### 3.2. Citicoline Usage in Cognitive Functions Betterment, Dementia Prevention, and Treatment

Cognitive functions are understood as being memory, attention, speech, awareness, and other, more complex functions, e.g., abstract thinking, judging, and calculation. These are essential functions in everyday situations. The decline of those skills due to aging, dementia, or brain damage is linked with increased hindrance in work and a self-reliant life. Due to an aging society, the problem of dementia will become increasingly prevalent and begin to impact society and its finances. Hence, it is crucial to research substances that can impede the negative effects of such processes. Another important aspect is the usage of such compounds to enhance natural abilities in school and work. The application of safe drugs that can improve learning and the ability to work could potentially impact society and contribute to its development.

During cognitive functions assessment, many scales and questionnaires are used. The Mini-Mental State Examination (MMSE) is a screen test to assess mental functions and dementia: the higher the score, the better preserved are cognitive functions. The correct results range from 27 to 30. The next scales are Activities of Daily Living (ADL) and Instrumental Activity of Daily Living (IADL). ADL is used to assess daily activities crucial for survival, such as hygiene and nutrition. IADL encompasses activities that are not essential for survival (finances, transportation), but they greatly enhance the self-reliance of individuals and raise the quality of life. A higher score in both scales attests to the better functioning of a person. The Neuropsychiatric Inventory (NPI) is used in the evaluation of typical neuropsychiatric symptoms, such as mood and behavior alteration, and stimuli perception. Higher scores in this scale are linked with a higher intensity of symptoms. The Geriatric Depression Scale (GDS) is used in depression assessment. The higher the score, the more severe the depression.

In the first cited paper, citicoline was combined with rivastigmine, and the effect of the combination was measured among mixed dementia (MD) and Alzheimer’s disease (AD) patients. Patients were tested at onset as well as in the 3rd and 9th month of the study using many scales: MMSE, ADL, IADL, NPI, and GDS. At the beginning of the study, there were no noticeable differences between groups on every scale. In the 3rd (*p* = 0.001) and 9th (*p* = 0.000) month, the MMSE results of citicoline users were significantly better (2 points on average) compared to the control group. NPI results proved to be better in the research group (7.12 vs. 9.51; *p* = 0.000). Results achieved in ADL, IADL, and GDS were not noticeably different between control and research groups [46]. Similar research was conducted by Controneo et al. [47]. In this research, patients had mild vascular dementia (MMSE ≥ 21). Significant differences in MMSE were not observed between the groups. However, there was a steep decline in the control group in comparison to citicoline users in the 3rd and 9th month of the study (in both, *p* = 0.0001). In the test group, there was no drop in MMSE results; the control group noticed a significant decline. ADL and IADL results were similar in both groups. However, GDS results were slightly better among citicoline users, albeit these results were not statistically significant (*p* = 0.06). Citicoline, despite not enhancing cognitive functions in this research, prevented dementia development.

In another study, citicoline was combined with an acetylcholinesterase inhibitor in Alzheimer’s disease patients. In this study, the group receiving citicoline achieved higher results in MMSE both in the 3rd month (16.88 vs. 17.62; *p* = 0.000) and in the 9th month (17.62 vs. 17.89; *p* = 0.000) of treatment. This research also did not record significant differences in ADL, IADL, NPI, and GDS between groups [48]. The next analyzed research regarded the citicoline effect on mild cognitive impairment in Parkinson’s disease. To assess the function level of patients, two scales were employed: Montreal Cognitive Assessment (MoCA) and Scales for Outcomes in Parkinson’s Disease-Cognition (SCOPA-COG). These are the scales used in the diagnosis of mild cognitive function impairment; the higher the score, the better functioning of the individual. At the onset of the study, there was no difference in MoCA and SCOPA-COG scores between the control and research groups. After 12 months of study, the results of the citicoline group were not significantly better than the control group (23.65 vs. 22.53; *p* > 0.05). However, in the 18th month, the discrepancy between results was bigger (23.12 vs. 21.49; *p* < 0.05). On the SCOPA-COG scale, results achieved by citicoline users were visibly better both at 12 months (21.55 vs. 20.73, *p* < 0.05) and 18 months (21.09 vs. 19.25, *p* < 0.01) of study. Citicoline did not improve results on both scales. Starting results in MoCA and SCOPA-COG were 24.03 and 23.79, respectively. However, citicoline did inhibit the progression of cognitive function impairment [49].

The pro-cognitive effects of citicoline and the slowing of the development of age-related brain lesions may be due to its action on the increase in phosphodiesters (PDE) levels in the brain. Healthy older patients participated in the study. After 6 weeks of taking the drug, an average 7.3% increase in brain PDE (including glycerophosphocholine (GPC) and glycerophosphoethanolamine (GPE)) was observed (*p* = 0.008). After 12 weeks, no significant increase in PDE levels was observed compared to those taking citicoline. Interestingly, the increase in PDE levels correlated with the improvement in verbal learning on the California Verbal Learning Test (CVLT) scale [15].

Alvarez et al. [50] assessed the effect of citicoline on cognitive functions and cerebral flow in the middle cerebral artery (MCA) in patients with Alzheimer‘s disease. The Alzheimer’s Disease Assessment Scale (ADAS) and the Clinical Interview Based Impression of Change (CIBIC) were used to assess cognitive functions. ADAS assesses basic cognitive functions; the higher the score, the worse the functioning. After 12 weeks, the difference between the groups in the CIBIS scores did not reach statistical significance. In contrast in the ADAS scale, the citicoline group achieved significantly better results (difference between groups 3.23 ± 1.8, *p* < 0.05). In cerebral flows in the citicoline group, a significant increase in mean systolic and diastolic velocities in MCA compared to the placebo group was observed (*p* < 0.05)

In a study by Spiers et al. [51], the effect of the drug on memory was assessed in healthy volunteers. The Logical Memory subtest stories of the Wechsler Memory Scale (WMS) and the Wechsler Memory Scale-Revised (WMSR) were used to assess memory. After 3 months, patients from the citicoline group with relatively inefficient memories had significantly better results in delayed logical memory test compared to baseline results (*p* < 0.05). Citicoline has not been shown to improve memory in people with efficient memory.

Cohen et al. [52] assessed the effectiveness of citicoline in people with vascular dementia. The drug was taken for a year. A battery of neuropsychological tests was used to assess neuropsychological functioning, consisting of a series of tests assessing various cognitive functions. Participants also had the MRI. There were no significant differences in neurocognitive change scores between baseline and either the 6- or 12-month assessments across any of the cognitive domains between the placebo and citicoline group (in all tests *p* > 0.05). The changes observed in the MRI also did not differ significantly between the groups (*p* = 0.17). Citicoline in this study proved unsuccessful.

Another study focused on citicoline impact on motor functions and attention among teenagers (13–18 years old). To assess motor functions, the Finger Tap Test was conducted. The Finger Tap Test (FTT) consists of the Finger Tap Total Dominant Hand (FTDH) and Finger Tap Total Non-Dominant Hand (FTNDH). In this test, the subject has to tap the button with a selected finger as fast as possible during a specific time. In attention evaluation, the Ruff 2&7 Selective Attention Test (RSAT), which consists of RSAT speed and RSAT accuracy, the Computerized Performance Test, Second Edition (CPT-II), divided into CPT-II detectability (ability to focus on computer tasks) and CPT-II commission errors (impulsiveness test), were used to evaluate attention. At the start of the study, no difference was observed in FTT, RSAT, and CPT-II between the control and test groups. Results in FTDH were higher in the test group in comparison to the control group after 28 days of citicoline supplementation (*p* = 0.03); however, differences in FTNDH did not reach statistically significant values (*p* = 0.62). In the RSAT speed test, the citicoline supplemented group also reached higher scores than the control group (*p* = 0.02), but in RSAT accuracy, the difference was small (*p* = 0.86). Compared to the start of the research, the citicoline users achieved significantly better results both in CPT-II detectability and CPT-II commission error (*p* = 0.03 and *p* = 0.01, respectively). Citicoline supplementation was linked with motor function enhancement, attention betterment, and impulsiveness reduction among test subjects [53].

The next study focused on citicoline combined with the effect of drinking on behavior control and executive functions. The Continuous Performance Task (CPT), which was used in the evaluation of concentration, memory, and impulse control, was used in the study. Other examinations used were the Austin Maze (hand–eye coordination and memory), Go/No-Go task (concentration, behavioral inhibition), and Digit Symbol Substitution test (attention, spatial coordination, and information processing). The last performed test was the Trail Making Test, which assessed information processing speed, brain plasticity, and executive functions. Subjects had an electroencephalography (EEG) taken in repose and during an examination. Thirty minutes after the drink was consumed, EEG results were taken. People who drank citicoline with caffeine solved the Austin Maze significantly faster than the control group (*p* = 0.008), and learned faster how to solve it (*p* = 0.008) (people from the test group solved the labyrinth in 134 s on average, whereas the control group averaged 186 s). The number of trials needed to solve the test perfectly was also significantly lower in the test group (*p* = 0.028). In CPT, people under the effect of citicoline had a faster reaction time (*p* = 0.001) and made fewer mistakes (*p* = 0.001) in comparison to the placebo group. In Digit Symbol Substitution tests, those solved by supplemented people had more positive answers (*p* = 0.008), and Go/No-Go had significantly fewer errors (*p* = 0.006) than the control group. However, there was no significant difference in the Trail Making Test between groups. Those results show that citicoline increases the capability to remember and improves concentration and perceptivity. There was increased brain activity in the frontal lobe and prefrontal cortex in the EEG measured by event-related potentials (P450) (*p* < 0.05). Patients who received citicoline had higher amplitude-measured potentials [54].

Bruce assessed the effect of a citicoline-containing drink on changes in the potentials recorded in the EEG during rest and performing an intellectual task (event-related potentials, ERPs). The alpha and gamma waves visible in the EEG were considered to be modifiable by attention and were therefore measured. The Auditory Oddball test was used as a task to assess awareness, in which the subjects were to ignore low-pitched sounds (500 Hz) and respond to high-pitched sounds (1000 Hz). The test was performed 30 min after drinking the citicoline drink. In ERPs, waves with two potentials were distinguished: N100 and N200. N100 waves are related to focus and arousal, whereas N200 waves are considered to be related to cognitive processes. People who received the citicoline drink had significantly higher alpha wave amplitudes (*p* < 0.05). In terms of gamma waves, no significant differences were found. In an event-related potential (ERP), a significant increase in N100 waves was noted compared to the placebo (*p* < 0.05). There were no significant differences in the N200 wave range. Citicoline, in this study, raised attention among healthy people [55].

Another study assessed the effects of citicoline on cognition with the CogState Battery. This test assesses, among others, memory, attention, and decision-making abilities. Based on the results obtained, 24 volunteers were divided into three groups: those with low results, average results, and high achievements, each consisting of eight people. Then, in each group, some people received a placebo, and some received citicoline, in either 500 or 1000 mg doses. In terms of psychomotor skills, in the group with low results, both 500 mg (*p* < 0.006) and 1000 mg (*p* < 0.001) of citicoline significantly improved the results compared to those receiving placebo. Citicoline did not affect the results obtained by the other groups. In terms of attention, citicoline did not significantly improve the results in any of the groups. Working memory was enhanced considerably in the low-score group, both at 500 mg (*p* < 0.008) and 1000 mg (*p* < 0.021); citicoline did not affect the other groups. In terms of problem-solving, citicoline also proved to be significantly beneficial in the low-score group, both at 500 mg (*p* < 0.005) and 1000 mg (*p* < 0.037). In terms of delayed memory, only in the group with low results did citicoline significantly improve the results obtained at the dose of 1000 mg (*p* < 0.042). In verbal memorization, the 1000 mg dose significantly improved the results of the group with a low initial result (*p* < 0.0001). In delayed oral memorization, citicoline at doses of both 500 and 1000 mg also improved the results of the group with low initial scores (*p* < 0.033 and *p* < 0.042, respectively). Interestingly, citicoline reduced the results obtained by the group with high initial scores significantly (*p* < 0.05) in all of the examined aspects. This study demonstrated that citicoline improved cognitive functions in people with low initial functioning levels, but it decreased these functions in people with high initial levels of functioning [56].

Another study examined the effect of citicoline on cognitive functions in people with somatoform disorders. Cognitive functions were assessed using the Cognitive Emotional Regulation Questionnaire (CERQ), and impulsivity and attention were assessed using the Test of Variables of Attention (TOVA). The tests were performed initially, after 30 days (at the end of citicoline administration), and after 60 days. On day 60 of treatment initiation, patients receiving citicoline achieved significantly better TOVA scores (*p* < 0.05) than those receiving placebo. They also outperformed the baseline (*p* < 0.05).

Similarly, on the CERQ scale, the results of the test group were significantly better than those of the control group (*p* < 0.05) in terms of long-term thinking, planning, and the ability to focus. Most of the aspects examined also showed significant improvement compared to the start of treatment (*p* < 0.05). To summarize, citicoline has been proven to be useful as a means of improving the cognitive skills and functioning of patients with somatoform disorders [57].

Citicoline, in almost all of the studies, has been found to be an effective drug in terms of its influence on cognitive functions. In patients with dementia of various origins, it inhibited the progression of the disease during observation and improved their daily functioning. It has also proved useful in improving the results achieved in tests assessing cognitive functions in healthy people. The most important features of research cited above are summarized in the table (Table 3).

### 3.3. Application of Citicoline among Patients after Traumatic Brain Injury (TBI)

Head injuries continue to be a significant cause of disability and mortality. In the United States, they are the leading cause of disability in people under 45 and account for 30.5% of all injury-related deaths [58]. The search for a treatment that would reduce the negative consequences of brain injuries poses a significant problem.

In the study by Trimmel et al. [59], 67 patients with brain trauma were administered citicoline during their stay in the intensive care unit (ICU). The Rotterdam CT score, Glasgow Coma Scale (GCS), and the Injury Severity Score (ISS) were used to initially assess the condition of patients after trauma. The state of patients at the start of treatment did not differ significantly in the two groups (*p* > 0.05). Mortality during the stay in ICU was 5% for the research group and 24% for the control group (OR 6.7, *p* < 0.01), and the mortality during hospitalization was 9% and 24% (*p* = 0.035), respectively. The 6-month mortality in the citicoline group was 13%, and it was 28% in the placebo group (*p* = 0.031). A share of 34% of patients in the study group had unfavorable results after 6 months, and 57% in the control group had unfavorable results after 6 months (*p* = 0.015). Patients from the citicoline group had significantly higher odds of ICU survival (OR 6.7, *p* < 0.01), hospital survival (OR 3.2, *p* = 0.024), favorable outcome after 6 months (OR 2.5, *p* < 0.01), and survival after 6 months (OR 2.6, *p* = 0.037). After adjustment for age, the first available GCS, and ISS, adjusted ORs still disclosed significantly better odds for ICU survivals (OR 6.7, 95% CI 1.6–28.8, *p* = 0.014) and favorable outcome after 6 months (OR 2.6, 95% CI 1.1–6.0, *p* = 0.022) in the citicoline group. Citicoline contributed to the reduction of both per-accident and long-term mortality. Moreover, it helped to achieve more favorable long-term treatment outcomes.

In another study, the influence of citicoline on the GCS score was assessed daily, and the levels of fetuin-A and Matrix Gla protein (MGP) were examined. Fetuin-A is an anti-inflammatory protein that inhibits the production of pro-inflammatory cytokines and the development of atherosclerosis. MGP also prevents the formation of atherosclerosis. During the observation, no significant differences in the obtained results in GCS were observed between the groups (*p* > 0.05). The level of fetuin-A did not differ significantly between the groups (*p* = 0.08 on the 6th and 12th days of the study). A significantly higher level of MGP was observed in the study group (*p* = 0.01) on the 12th day of observation. Citicoline in this study showed moderate effectiveness—it had no significant effect on GCS. It only influenced the level of MGP [60].

In the multicenter study, the Citicoline Brain Injury Treatment Trial (COBRIT), 607 patients were administered citicoline for 90 days. The TBI Clinical Trials Network Core Battery was used to assess patient function. This is a complex test, consisting of nine components, in which the overall level of functioning, including intelligence and memory, is determined. The two groups did not differ in the 90-day evaluation with respect to the TBI Clinical Trials Network Core Battery (OR 0.98, 95% CI 0.83–1.15). Moreover, the study included the Glasgow Outcome Scale–Extended (GOS-E) to assess the degree of recovery of head injury victims. After 90 days from the start of treatment, the mean improvement in the GOS-E score in the research group was 35.4%, and in the control group, it was 35.6%. On the other scales, the improvement ranged from 37.3% to 86.5% in the citicoline group and from 42.7% to 84.0% in the control group (*p* > 0.05 for all). Citicoline results were not significantly different compared to those of the control group. Similarly, in the study at 180 days after the start of treatment, the results in both groups did not differ significantly (OR 0.87, 95% CI 0.72–1.04, *p* = 0.13). Moreover, among patients with complicated mild brain trauma, patients in the placebo group achieved substantially better results in the studies than in the research group (OR 0.72, 95% CI 0.56–0.91, *p* = 0.004). In patients with moderate/severe TBI, no statistically significant difference was observed between groups (OR 1.26, 95% CI 0.92–1.70, *p* = 0.14). In addition, the survival rates in the first 30 days did not differ between groups (*p* = 0.17) [61].

A total of 2706 patients from twelve controlled-trials were included in the meta-analysis, which was performed by Secades [62]. To be included, the trials must assess the effect of citicoline in the acute phase of TBI, be comparative studies, and have independence outcomes evaluated with the Glasgow Outcome Scale (GOS) or similar scales. Articles from the last four decades were analyzed. The primary efficacy measure was patient independence at the end of a follow-up period, which was evaluated as a score GOS 4–5 indicating a perfect outcome or with mild sequelae. According to the formal meta-analysis, based on the random effects model, the use of citicoline is associated with a significant increase in the rates of independence with an OR of 1.815 (95% CI 1.302–2.530). Due to the time gap of 34 years between studies, a significant heterogeneity (*p* = 0.001) was detected. Under the fixed-effects model, the meta-analysis attains an OR of 1.451 (95% CI 1.224–1.721), reinforcing the results obtained. Importantly, the effectiveness of citicoline has decreased over the years due to improvements in the overall quality of healthcare. However, citicoline should still be added to treatment in people after TBI.

The effectiveness of citicoline in TBI treatment is not entirely clear. In a large, multicenter study, its effectiveness was comparable to that of a placebo. However, the clinical effectiveness of citicoline has been proven in performed meta-analysis. Hence, continued exploration of the use of citicoline and other drugs in the treatment of brain trauma is advisable. The most important features of the research cited above are summarized in the table (Table 4).

### 3.4. The Application of Citicoline in the Regeneration of Peripheral Nerves after Injury and the Treatment of Neuropathic Pain: Animal Models

Various pathological conditions can lead to damage to peripheral nerves and the occurrence of neuropathic pain. One of the most common causes is degenerative changes leading to injury, e.g., the sciatic nerve, diabetes-related neuropathy, and drug-induced nerve damage. Many polyneuropathies of various etiologies also lead to nerve damage. With the aging of the population, the problem of nerve damage will become more frequent and require new treatments. To date, the use of citicoline has been studied in this neurological condition only in animal models.

In the study by Emril et al., the effect of a gelatin sponge soaked with various concentrations of citicoline on the regeneration of the damaged sciatic nerve was investigated. Different amounts of citicoline (100 µmol/L) were used in two groups: 0.4 and 0.8 mL. The Sciatic Functional Index (SFI) and Extensor Postural Thrust (EPT) were used to assess the motor functions of the nerve. Von Frey filaments (threshold 100 g) were used to assess neuropathic pain. After 4 weeks, neuropathic pain only occurred in 2 of 10 rats in the 0.4 mL citicoline group and 8/10 in the control group (*p* < 0.05). In the 0.8 mL group, 4/10 rats suffered from neuropathic pain (*p* = 0.18). There were no significant differences between the groups in the SFI test (*p* = 0.26). In the EPT test, the groups receiving citicoline had a significantly lower percentage of a motor deficit than in the control group: for 0.4 and 0.8 mL, respectively, 14.28% and 20.6% (*p* = 0.00). Citicoline in this test was effective in alleviating neuropathic pain and reducing motor deficit [63].

In another study, rats with injured sciatic nerves received citicoline at three different doses: 300, 600, and 900 mmol/kg. The SFI was used to assess the improvement in function, and the assessment was made at 4, 8, and 12 weeks. An electromyographic examination (EMG) was also performed at week 12. Thereafter, the nerves were analyzed histologically. In the SFI test, citicoline at a dose of 900 mmol/kg resulted in a significant improvement in the function of the sciatic nerve at both 8 and 12 weeks compared to the control group and at week 8 compared to the other study groups (*p* < 0.05). Citicoline at a dose of 600 mmol/kg caused a significant improvement at 12 weeks of follow-up (*p* < 0.05). In addition, in EMG at week 12, citicoline at doses of 600 and 900 mmol/kg caused a significantly smaller delay compared to the control group. In the microscopic examination, rats in the 900 mmol/kg citicoline group had a considerably denser axonal network and significantly more myelinated axons compared to the other groups (*p* < 0.001). In the study groups, the scar after injury was substantially smaller than in the control group (*p* < 0.05) [64].

In the next study, rats after sciatic nerve injury were divided into five groups: control, receiving citicoline, cytidine, choline, and cytidine + choline. A portion of the rats in each group had sutured nerves on day 1 and some on day 3 post-cut. The SFI examination was performed 4, 8, and 12 weeks after the operation. After 12 weeks, the macro and microscopic examination of the nerve was performed. The citicoline group at weeks 4, 8, and 12 had significantly better results in the SFI test than the control group, and results were comparable to the cytidine + choline group (*p* < 0.001). In the macroscopic examination, rats with nerve repairs on day 1 receiving citicoline had significantly better nerve regeneration. The nerve repaired rats achieved similar results in all groups after 3 days. In histology, rats receiving citicoline had significantly more axons, which were better organized and larger in diameter than those of rats in the control group. Histological observations were related to the SFI score (*p* < 0.01) [65].

In a study by Caner et al., in which the same substances were tested, similar results were obtained in the SFI test, and in the macro and microscopic examination of the sciatic nerve. In addition, EMG was performed in this experiment. The citicoline group had a significantly higher amplitude of the muscle response than the control group (*p* < 0.001) [66].

In a study by Özay et al. [67], the effectiveness of citicoline in accelerating the regeneration of the damaged sciatic nerve was also assessed. For this purpose, SFI and EMG were used, and the macro and microscopic evaluation of the nerve was performed. Rats received 0.4 mL (100 µmol/L) citicoline or 0.4 mL placebo. The SFI values as an indicator of functional recovery were significantly better in rats treated with citicoline than those in rats treated with saline. A statistical difference between the two groups was found in every assessment starting from 4 weeks post surgery (in 4, 8, and 12 weeks *p* < 0.001). In EMG, no significant difference between groups was observed 4 weeks after surgery. However, after 12 weeks, nerve action potentials in the citicoline group were significantly higher (*p* < 0.05). In microscopic evaluation, nerves treated with citicoline had significantly higher axon counts and mean axon diameters than nerves treated with saline (*p* < 0.05). Citicoline in this study also improved nerve regeneration. Kanat et al. studied the influence of citicoline on the occurrence of neuropathic pain induced by oxaliplatin (OXA). To assess the neuropathic pain threshold, the Randall–Sellito test was used, which consisted of compressing the animal’s paw with increasing weight. After OXA administration, the pain threshold decreased from 144.3 to 66 g on the first day and 47.5 g on the second day after administration. Citicoline in the dose of 2 µmol increased the pain threshold to 150 g, and this effect lasted for several hours (*p* < 0.001). Lower doses also increased the pain threshold but showed a shorter duration of action [68].

In another experiment, the influence of citicoline on the inflammatory pain process induced by Carrageenan injection and on neuropathic pain caused by damage to the sciatic nerve was investigated. The Randall–Sellito test was used to assess the pain threshold. In inflammatory pain, the dose of 2 µmol increased the sensory threshold pain from 50 to 300 g (*p* < 0.001). In neuropathic pain, the pain threshold changed from 75 to 250 g (*p* < 0.001) [69].

Another experiment investigated the effect of citicoline on the level of metalloproteinases after sciatic nerve injury. Metalloproteinases are enzymes responsible for the destruction of Schwann cells, which are responsible for the regeneration of neuronal axons after nerve damage. Their level correlates with the ability to regenerate damaged nerves. Sciatic nerve samples for testing the level of metalloproteinases type 2 and 9 were taken on days 1, 3, and 7 after the nerve injury. It was observed that on day 1 after surgery, the activity of metalloproteinases 2 and 9 increased to a similar extent in the control group and that receiving citicoline. However, on the 3rd and 7th day after surgery, the activity of metalloproteinase 2 and 9 in the group receiving citicoline decreased by 36% and 23%, respectively, and by 15% and 12% in the control group, respectively. Moreover, histological examination showed significantly more myelinated axons in the citicoline group than in the control group: 34.5%, 36%, and 90.4% more, respectively, on days 1, 3, and 7 (*p* < 0.001). The total number of myelinated axons in the citicoline group was 75%, 183%, and 146% greater on days 1, 3, and 7, respectively (*p* < 0.001). The influence of citicoline on the level of metalloproteinases may explain its beneficial effect in the regeneration of damaged peripheral nerves [70].

Citicoline is beneficial in nerve regeneration and the reduction of neuropathic and inflammatory pain in most of the studies cited above. It resulted in faster and more intense nerve regeneration, which is visible both in the macroscopic and microscopic images. It also increased the density of the axonal network. Its beneficial effect may be related to including the reduction of the concentration and activity of metalloproteinases.

## 4. Strengths and Limitations

The main strength of this article is a comprehensive look at the use of citicoline in neurology. Articles exploring the use of citicoline in various neurological conditions were included. Articles from different years and databases were reviewed.

The main limitation/bias of our review is to include only articles in the English language. Another limitation is the lack of access to the full content of some articles.

## 5. Conclusions

This systematic review showed that citicoline has a wide range of uses in neurological conditions. In dementia, it is useful primarily in inhibiting disease progression, and, according to the results of some studies, reversing adverse changes. Citicoline also improved memory and other cognitive functions among healthy volunteers. For this purpose, they were assessed with various tests, which adds credibility to these studies. Citicoline has also been shown to be a promising drug in reducing neuropathic pain and accelerating nerve regeneration. Unfortunately, these studies were only conducted in animal models. Citicoline may prove to be a potentially beneficial adjunct in the treatment of stroke. However, citicoline has unclear effects in the treatment of brain injuries. Citicoline, depending on its application, can be considered both as a dietary supplement and as a medicine. Further research on this substance should be carried out, including other neurological and non-neurological diseases.

## Figures and Tables

**Table 1:** Inclusion and exclusion criteria of articles.

Inclusion Criteria	Exclusion Criteria
Written in English	Articles written in a language other than English
Clinical trial, multicenter study, meta-analysis	Review, case report
Studies on animals and humans	Studies other than on animals and humans

**Table 2:** The most important features of research based on patients after a stroke event.

References	Number of Patients Received Citicoline	Mean Age (Years)	Dose of Citicoline (mg/Daily)	Period of Citicoline Administration (Days)	Used Methods to Assessment Effectiveness of Citicoline	Frequency of Examination by Scales (Days after Start Treatment)
Mehta et al. [24]	20	59.5	500 × 2	42	NIHSS, BI	0, 11, 90
Ghosh et al. [25]	50	64.02	1000 × 2500 × 2	5 and 25	BI	30, 90
Alvarez-Sabín et al. [26]	172	67.2	1000	365	mRS	30, 180, 365
Jiménez et al. [27]	86	68.6	1000, 500	9	NIHSS, mRS	30, 90
Alvarez-Sabín et al. [28]	86	67.5	1000	365	EuroQol-5D	30, 180, 365, 2 years
Cho et al. [29]	4191	67.04	500–2000	42 and 84	s-NIHSS, s-BI, mRS	42, 84
Clark et al. [30]	195	67.5	500, 1000, 2000	42	BI, NIHSS, mRS	7, 21, 42, 84
Secades et al. [31]	19	74.5	2000	14	mRS, NIHSS	14, 90
Martynov et al. [32]	89	62.7	1000	21	SSS, BI, mRS	1, 7 and 21 (SSS); 21–24 (BI, mRS)
Iranmanesh et al. [33]	16	61.15	500	14	Lovett scale	90
Tazaki et al. [34]	133	-	1000	14	JCS, GIR	1, 2, 3, 7, 14
Sobrino et al. [35]	26	71.4	2000	42	NIHSS, mRS	7, 90
Tykhomyrov et al. [36]	33	76	1000	14	-	-
Zang et al. [37]	52	57.53	500	28	NIHSS, BI	28
Seifaddini et al. [38]	32	-	500	7	-	-
Dávalos et al. [40]	1148	72.9	2000 × 21,000 × 2	3 and 39 (42 overall)	NIHSS, BI, mRS	90
Mittall et al. [41]	24	54.83	500 × 2	42	NIHSS, mRS	90
Clark et al. [43]	267	70	500	42	NIHSS, BI, mRS	7, 21, 42, 84
Clark et al. [44]	453	68	2000	42	NIHSS, mRS	90
Warach et al. [45]	41	68.5	500	42	BI, NIHSS, mRS, MRI	7, 42, 84

Barthel Index (BI); National Institute of Health Stroke Scale (NIHSS); modified Rankin Scale (mRS); Scandinavian Stroke Scale (SSS); The Japanese Coma Scale (JCS); Global Improvement Rating(GIR); magnetic resonance imaging (MRI).

**Table 3:** Key clinical features of studies in dementia patients and healthy volunteers.

References	Number of Patients Received Citicoline	Mean Age (Years)	Dose of Citicoline (mg/Daily)	Period of Citicoline Administration (Days)	Used Methods to Assessment Effectiveness of Citicoline	Frequency of Examination by Scales (Days after Start Treatment)
Castagna et al. [46]	92	81.3	1000	270	MMSE, ADL, IADL, NPI, GDS	90, 270
Cotroneo et al. [47]	265	79.9	500 × 2	270	MMSE, ADL, IADL, GDS	90, 270
Gareri et al. [48]	251	-	1000	270	MMSE, ADL, IADL, NPI, GDS	90, 270
Zhenguang et al. [49]	41	61.7	200 × 3	18 months	MoCA, SCOPA-COG	12, 18 months
Alvarez et al. [50]	13	73	1000	84	ADAS	84
Babb et al. [15]	19	70.3	500	42, 84	CVLT	42, 84
Spiers et al. [51]	46	67.2	1000	90	WMS, WMSR	30, 90
Cohen et al. [52]	15	78.1	1000			180, 360
McGlade et al. [53]	51	15.41	250/500	28	FTT, RSAT, CPT-II	28
Bruce et al. [54]	30	24.2	250	-		30 min
Bruce et al. [55]	10	28.1	250	-	EEG	30 min
Knott et al. [56]	24	21.3	500/1000	-	Cogstate	12-14
Chutko et al. [57]	46	32.3	1000	30	TOVA, CERQ,	30, 60

Mini-Mental State Examination (MMSE); Activities of Daily Living (ADL); Instrumental Activity of Daily Living (IADL); The Neuropsychiatric Inventory (NPI); Geriatric Depression Scale (GDS); Montreal Cognitive Assessment (MoCA); Scales for Outcomes in Parkinson’s Disease-Cognition (SCOPA-COG); The Alzheimer’s Disease Assessment Scale (ADAS); the California Verbal Learning Test (CVLT); the Wechsler Memory Scale (WMS); the Wechsler Memory Scale-Revised (WMSR); The Finger Tap Test (FTT); Ruff 2&7 Selective Attention Test (RSAT); Computerized Performance Test, Second Edition (CPT-II); electroencephalography (EEG); Test of Variables of Attention (TOVA); Cognitive Emotional Regulation Questionnaire (CERQ).

**Table 4:** Key clinical features of studies in patients after traumatic brain injury (TBI).

References	Numer of Patients Received Citicoline	Mean Age (Years)	Dose of Citicoline (mg/Daily)	Period of Citicoline Administration (Days)	Used Methods to Assessment Effectiveness of Citicoline	Frequency of Examination (Days after Start Treatment
Trimmel et al. [59]	67	54.6	3000	Treatment in ICU, maximally 21 days	Mortality, unfavorable outcome	Hospital discharge and 180 days
Shokouhi et al. [60]	29	30.94	2000	15	GCS, level of fetuin-A, MGP	6, 12, 15
Zafonte et al. [61]	607	-	2000	90	TBI Clinical Trials Network Core Battery	30, 90, 180

Glasgow Coma Scale (GCS); Matrix Gla protein (MGP).
